# Improvement of retinal function in Alzheimer disease-associated retinopathy by dietary lysophosphatidylcholine-EPA/DHA

**DOI:** 10.1038/s41598-023-36268-0

**Published:** 2023-06-06

**Authors:** Dhavamani Sugasini, Jason C. Park, J. Jason McAnany, Tae-Hoon Kim, Guangying Ma, Xincheng Yao, Babu Antharavally, Anil Oroskar, Asha A. Oroskar, Brian T. Layden, Papasani V. Subbaiah

**Affiliations:** 1 Division of Endocrinology, Diabetes, and Metabolism, Department of Medicine, University of Illinois, Chicago, IL 60612 USA; 2 Department of Ophthalmology and Visual Sciences, University of Illinois, Chicago, IL 60612 USA; 3 Department of Biomedical Engineering, University of Illinois, Chicago, IL 60607 USA; 4 Orochem Technologies, Inc, Naperville, IL 60563 USA; 5 Jesse Brown VA Medical Center, Chicago, IL 60612 USA

**Keywords:** Biochemistry, Neuroscience, Diseases, Neurology, Pathogenesis, Risk factors

## Abstract

Alzheimer disease (AD) is the most prevalent cause of dementia in the elderly. Although impaired cognition and memory are the most prominent features of AD, abnormalities in visual functions often precede them, and are increasingly being used as diagnostic and prognostic markers for the disease. Retina contains the highest concentration of the essential fatty acid docosahexaenoic acid (DHA) in the body, and its deficiency is associated with several retinal diseases including diabetic retinopathy and age related macular degeneration. In this study, we tested the hypothesis that enriching retinal DHA through a novel dietary approach could ameliorate symptoms of retinopathy in 5XFAD mice, a widely employed model of AD. The results show that 5XFAD mice have significantly lower retinal DHA compared to their wild type littermates, and feeding the lysophosphatidylcholine (LPC) form of DHA and eicosapentaenoic acid (EPA) rapidly normalizes the DHA levels, and increases retinal EPA by several-fold. On the other hand, feeding similar amounts of DHA and EPA in the form of triacylglycerol had only modest effects on retinal DHA and EPA. Electroretinography measurements after 2 months of feeding the experimental diets showed a significant improvement in a-wave and b-wave functions by the LPC-diet, whereas the TAG-diet had only a modest benefit. Retinal amyloid β levels were decreased by about 50% by the LPC-DHA/EPA diet, and by about 17% with the TAG-DHA/EPA diet. These results show that enriching retinal DHA and EPA through dietary LPC could potentially improve visual abnormalities associated with AD.

## Introduction

Alzheimer disease (AD) is the most prevalent form of dementia in the elderly, affecting over 40 million people worldwide. Because of the aging demographics, AD is projected to affect over 120 million people by 2050. Although the most common clinical feature of AD is the impairment of cognitive functions and memory, abnormalities in visual function have also been reported by several studies^1–4^. In fact, the visual disturbances are often seen earlier than the cognition symptoms^3^. The accumulation of amyloid β, the major pathological marker associated with AD, often occurs in the retina earlier than in the brain^3,5^, and is therefore used as an early diagnostic marker for the disease. Because of its non-invasive accessibility, and its common developmental link to the brain, the retina is viewed as a window to the brain, and is increasingly being employed not only for the diagnosis of AD but also to monitor therapeutic potential of anti-AD drugs^6–8^.

A common feature of brain and retina is the presence of exceptionally high concentration of docosahexaenoic acid (DHA), the omega 3 fatty acid which has been shown to be essential for the normal development and function of both these organs^9,10^. Up to 60% of the total fatty acids in the rod outer segment membrane phospholipids has been reported to be DHA^11^, indicating a functionally significant role. The deficiency of DHA is associated with various ocular diseases, including retinitis pigmentosa^12^, diabetic retinopathy^13–15^, age-related macular degeneration^16^, dry eye disease, and glaucoma^13^. Although both epidemiologic^13,16^ and pre-clinical studies suggested beneficial effects of dietary DHA for retinal health, controlled clinical trials with the currently available supplements showed no significant benefit for retinopathy^17–19^, age-related macular degeneration^20,21^ or glaucoma^22^, although a combination of DHA and citicholine, a precursor of phospholipids, was reported to improve symptoms in a small group of glaucoma patients^22^.

The reason for the failure of the vast majority of the studies may be the special mechanism by which retina acquires DHA from the plasma. Unlike other tissues, brain and retina acquire DHA through a specific transporter called Mfsd2a which transports plasma DHA in the form of lysophosphatidylcholine (LPC), but not as triacylglycerol (TAG) or free fatty acid^23–25^. The deficiency of this transporter leads to several diseases of the brain and retina, supporting the need to supply DHA in the form of LPC^25^. Furthermore, overexpression of Mfsd2a in the eye was reported to decrease retinal neovascularization and vascular leakage in mouse models of retinopathy^26^. We have recently demonstrated that retinal DHA can indeed be increased efficiently in normal adult mice with dietary LPC-EPA/DHA^27^. Therefore, in this study, we tested the hypothesis that the retinal DHA is decreased in AD, and that increasing the retinal DHA through dietary LPC-DHA would prevent or ameliorate the retinopathy associated with AD.

## Materials and methods

### Animals

All animal protocols have been approved by the UIC animal care committee, and all methods were carried out in accordance with the relevant regulations and guidelines. The reporting of data here is in accordance with the ARRIVE guidelines. Male 5XFAD mice without the retinal degeneration allele (Pde6b^rd1^) were purchased from Jackson Labs (Bar Harbor, Maine, USA) and bred with female c57BL/6J mice in our facility. The offspring were genotyped using the Transnetyx genotyping services (Cordova, TN, USA) and the female mice hemizygous for the 5XFAD transgene and their non-transgenic WT littermates were used for the study. One month old 5XFAD mice were randomly divided into 3 groups and fed the following 3 types of modified AIN93G diet: (a) Control diet containing 7% fat by weight as corn oil; (b) LPC-diet, which contained 2.64 g omega 3 FA (EPA + DHA)/g in the form of LPC and 7% total fat made up with corn oil; and (c) TAG-diet, which contained 2.64 g omega 3 FA (EPA + DHA)/kg in the form of TAG, made up to 7% fat with corn oil. The WT mice were fed only the control diet (containing only corn oil with no added omega 3 FA). The animals were housed (5 per cage) in humidity controlled rooms maintained at 22 ± 1 C, with 12 h light/dark cycle. At 3 months of age (2 month feeding of special diets), ERG and OCT measurements were performed as described below. Groups of mice were sacrificed at 3, 6, 9, and 12 months of age for the analysis of retinal fatty acids by GC/MS, and amyloid measurement by ELISA.

### Diet preparations

LPC-EPA/DHA was prepared by the treatment of krill oil with lipase (28), followed by purification by column chromatography. Briefly, krill oil, 80 g (Superba Boost™, Aker BioMarine) was dissolved in 1.05 L of 95% (v/v) ethanol in water by stirring at room temperature for 30 min and the resulting solution was centrifuged for 10 min to remove the suspended particles. An immobilized acrylic lipase (80 g) from *Candida Antarctica B* (Strem Chemicals, Newburyport, MA.), was added to the supernatant solution, purged with nitrogen and incubated at 50 °C for 24 h in a metabolic shaker in dark. The digested suspension was centrifuged and the supernatant was separated from the immobilized enzyme. The digested solution was subjected to chromatography on a column packed with a polymeric hydrophobic resin, (OR32 resin, Orochem Technologies Inc.). The column, which was maintained at 50 °C, was first equilibrated with 4 column volumes of 80% ethanol in water at 300 ml/min. The digested krill oil was diluted to 80% ethanol in water, filtered and loaded on to the column. Elution was carried out with 80% ethanol in water and 1 L fractions were collected. Aliquots of the fractions were subjected to HPLC analysis using an Agilent 1100 series 3 μM C18 column (4.6 × 150 mm, Orochem Technologies Inc.) with the following parameters: temperature: 50 °C; flow rate: 1 mL/min; mobile phase: gradient of ethanol and water from 80% ethanol to 100% ethanol. Peaks were monitored at 215 nm. Fractions corresponding to LPC-EPA/DHA standard were pooled, concentrated by rotovap and stored at − 20 °C. Based on the peak area, LPC-EPA/DHA constituted 85% of the total lipid in the preparation, the rest being free fatty acids and neutral lipids. There was no PC or PE left after the lipase treatment.

An aliquot of the preparation was subjected to TLC separation on a silica gel plate, and the LPC spot was analyzed for its fatty acid composition by GC/MS^28^.The major fatty acids in LPC were: EPA (54%), DHA (25%), 18:1 (11%), 18:2 (5%) and 20:4 (2%). The LPC preparation containing 2.64 g EPA + DHA per kg diet was mixed with corn oil (67 g/g) and incorporated into AIN93G diet, and vacuum-packed by Dyets Inc. (Bethlehem, PA). The total fat in the diet was 7% by weight. The TAG diet contained Menhaden oil containing 2.64 g of EPA + DHA/g in the form of TAG and corn oil was added to bring the total fat to 7% by weight. The control diet contained 7% corn oil as the sole fat source. The diets were stored at − 20 C and thawed weekly, as needed.

### ERG analysis

Mice were dark-adapted for 2 h prior to testing. Anesthesia was administered by intraperitoneal injection of ketamine (100 mg/kg) and xylazine (5 mg/kg) under dim red illumination. Pupils were fully dilated with tropicamide (1%) eye drops. Stimuli were generated and delivered using a Celeris rodent electrophysiology system (Diagnosys LLC, Lowell, MA). ERGs were recorded using combined stimulator/electrode probes that were placed in contact with each cornea and aligned with the center of the pupils. Stimuli were delivered to one eye at a time, with the fellow, unstimulated eye serving as the reference. The flash stimuli consisted of brief (≤ 4 ms), achromatic, full-field luminance that ranged from − 3.0 to 1.5 log cd*s*m^−2^. A minimum of three single flash responses for each stimulus retinal illuminance were obtained and averaged for analysis. From these mean responses, the a- and b-wave amplitudes and implicit times were calculated according to convention^29^.

### OCT analysis

For OCT imaging, the mice were anesthetized with a mixture of ketamine (100 mg/g) and xylazine (5 mg/g) via Intraperitoneal injection. The pupil of the test eye was dilated with 0.5% tropicamide. When the pupil was fully dilated, the mouse was transferred to an adjustable stage. Lubricant eye gel (GenTeal, Alcon Laboratories. Fort Worth, TX) was applied to the eyes with a cover glass on top to prevent drying of the cornea. A custom-constructed spectral domain optical coherence tomography (SD-OCT) system^30^ was employed for the in vivo imaging. The lateral and axial resolutions were estimated at 12 µm and 3 µm, respectively. Three-dimensional OCT images were acquired around the optic nerve head (ONH) area with a diameter of 1.5 mm (Supplemental Fig. 1). To avoid the retinal thickness variation caused by the location bias, an OCT circular B-scan was reconstructed by selecting the OCT A-lines 0.65 mm away from the ONH center (Supplemental Fig. 1A). The retinal OCT images were segmented and measured via MATLAB software. The inner limiting membrane (ILM), the lower boundary of the outer plexiform layer (OPL), and the retinal pigment epithelium and Bruch’s membrane (RPE/BM) were segmented (Supplemental Fig. 1B). The thickness of the inner retina was measured from ILM to the lower boundary of OPL, the outer retina thickness was measured from the lower boundary of OPL to the lower boundary of RPE/BM, and the whole retina thickness was measured from ILM to the lower boundary of RPE/BM.

### Fatty acid analysis

At the indicated time points, the mice were anesthetized with ketamine-xylazine as above and the blood was drawn by cardiac puncture, using a heparinized syringe. The mice were perfused transcardially with cold PBS, pH of 7.4, the retinas were dissected and snap frozen in liquid nitrogen and were stored at − 80 °C.

Total lipids of the retina were extracted from the frozen retina samples by the Bligh and Dyer procedure^31^ and the fatty acid composition of the lipids was analyzed by GC/MS using a Supelco Omegawax column attached to Shimadzu QP2010 SE, as described previously^32^. Total ion current in the range of 50–400 m/z was used for quantitation of methyl esters, and the concentration of each fatty acid was calculated as the percent of total fatty acids.

### Amyloid B assay

Soluble and insoluble amyloid beta levels in the retina extracts were quantified using commercial ELISA kit (KMB3441; Invitrogen, Waltham, MA, USA). For soluble amyloid beta (Aβ) extraction, both the retinas from each mouse were pooled and homogenized in lysis buffer. The homogenate was spun for 50 min at 100,000× *g*, and the supernatant was used for the determination of soluble Aβ. The pellet was sonicated in 6 M guanidine hydrochloride in 50 mM Tris–HCl, incubated at 25 °C for 60 min, and centrifuged for 20 min at 170,000× *g*. The supernatant from this spin was used for determination of insoluble Aβ. Total protein concentration was determined using a BCA protein assay kit (23,225, Thermo Fisher Scientific, Waltham, MA, USA). Standard curves of Aβ42 were determined using the provided standards by the manufacturer.

### Statistical analyses

Statistical significance of differences in the fatty acid composition was determined by one-way ANOVA with Tukey post hoc correction for multiple comparisons (Prism software by Graphpad). For the ERG values, the extracted individual waveforms were compared employing GEE (generalized estimating equation) a population averaged model^29,33^. Statistical analyses were performed by using R (version 4.0.2; R Core Team, Vienna, Austria) and the two-sided p values were corrected for multiple comparisons by the Bonferroni method. Since there were 6 pairwise comparisons, the values < 0.0083 (0.05/6) were taken as significant.

## Results

### Time course of fatty acid changes in retina

**Figure 1:**
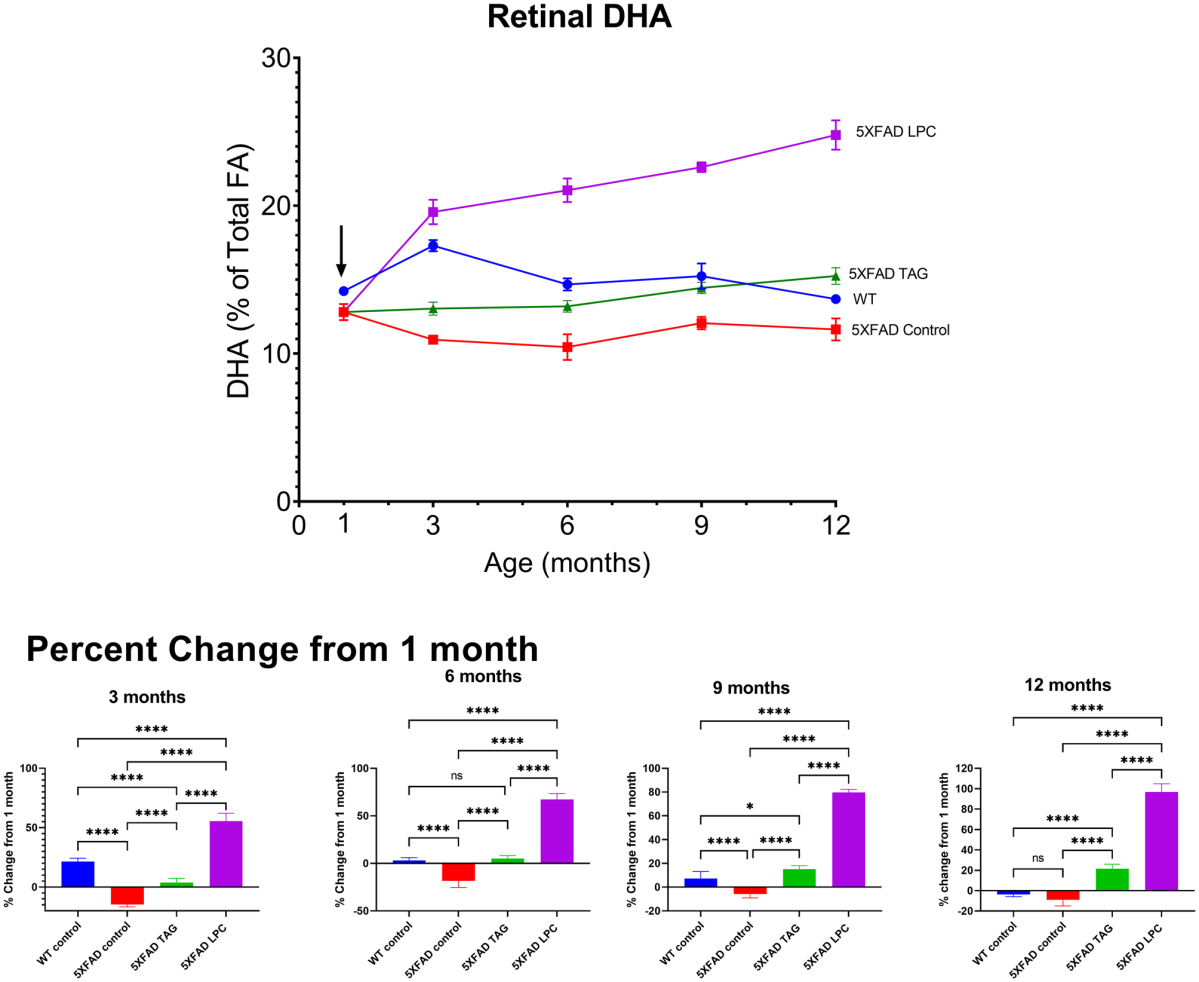
Time course of DHA incorporation into retinal lipids. One month old 5XFAD mice were divided into 3 groups and fed the following diets: (**a**) control diet which consists of AIN93G diet with 7% (w/w) corn oil as the sole source of fat; (**b**) AIN93G diet enriched with fish oil containing 2.64 g EPA + DHA in the form of TAG, made up to a total fat of 7% (w/w) with corn oil (TAG-diet) and (**c**) AIN93G diet enriched with 2.64 g EPA + DHA in the form of LPC, and made up to a total fat of 7% (w/w) with corn oil (LPC-diet). The WT littermates were fed control diet (**a**) which had no added omega 3 fatty acids. Six mice from each diet group were sacrificed at the indicated time points, the total lipids extracted from retina, and analyzed for the fatty acid composition as described in the text. The upper panel shows the %DHA in total lipids at various time points (mean ± SD, n = 6). The arrow represents the start of the experimental diets. The lower panel shows the percent changes of DHA from the baseline (1 month). Statistical significance was determined one way ANOVA analysis, and the *p* values were corrected for multiple comparisons by Tukey post hoc analysis. *p < 0.05;**p < 0.005, ***p < 0.005, ****p < 0.0001.

Groups of 5XFAD mice treated with one of the 3 types of diet and their wild type littermates on control diet were sacrificed at ages 1, 3, 6, 9, and 12 months (0, 2, 5, 8, and 11 months on the diets), and the retinas were analyzed for the fatty acid composition by GC/MS as described in Methods. The time course of incorporation of DHA into retina is shown in Fig. 1. The top panel shows the % DHA in each group at various time points, whereas the bottom panel shows the statistical analysis (one way ANOVA) of the percent changes from the baseline (1 month). At the age of 1 month, before the start of the experimental diets, the 5XFAD mice showed significantly lower % of retinal DHA, compared to their WT littermates (14.23 ± 0.31 in WT and 12.81 ± 0.54 in 5XFAD, *p* < 0.001) suggesting that the acquisition of DHA from the mother during gestation and lactation periods may be impaired. To our knowledge, a decrease in retinal DHA has not been reported previously in 5XFAD mice. The two omega 3 supplements differed significantly in their effects on retinal DHA, which increased by over 50% by 3 months (2 months of feeding) with the LPC diet, whereas it was increased by less than 2% by the TAG diet (Fig. 1, lower panel). At this time point, the DHA level in the LPC-fed group (19% of total) exceeded that of the WT group (17.3%), showing a complete normalization of DHA after 2 month feeding. In contrast, the TAG-fed group did not reach the WT levels until 9 months. By the age of 12 months (11 months of feeding) the retinal DHA was increased over 90% with the LPC diet, whereas the TAG diet increased retinal DHA by about 30%.

**Figure 2:**
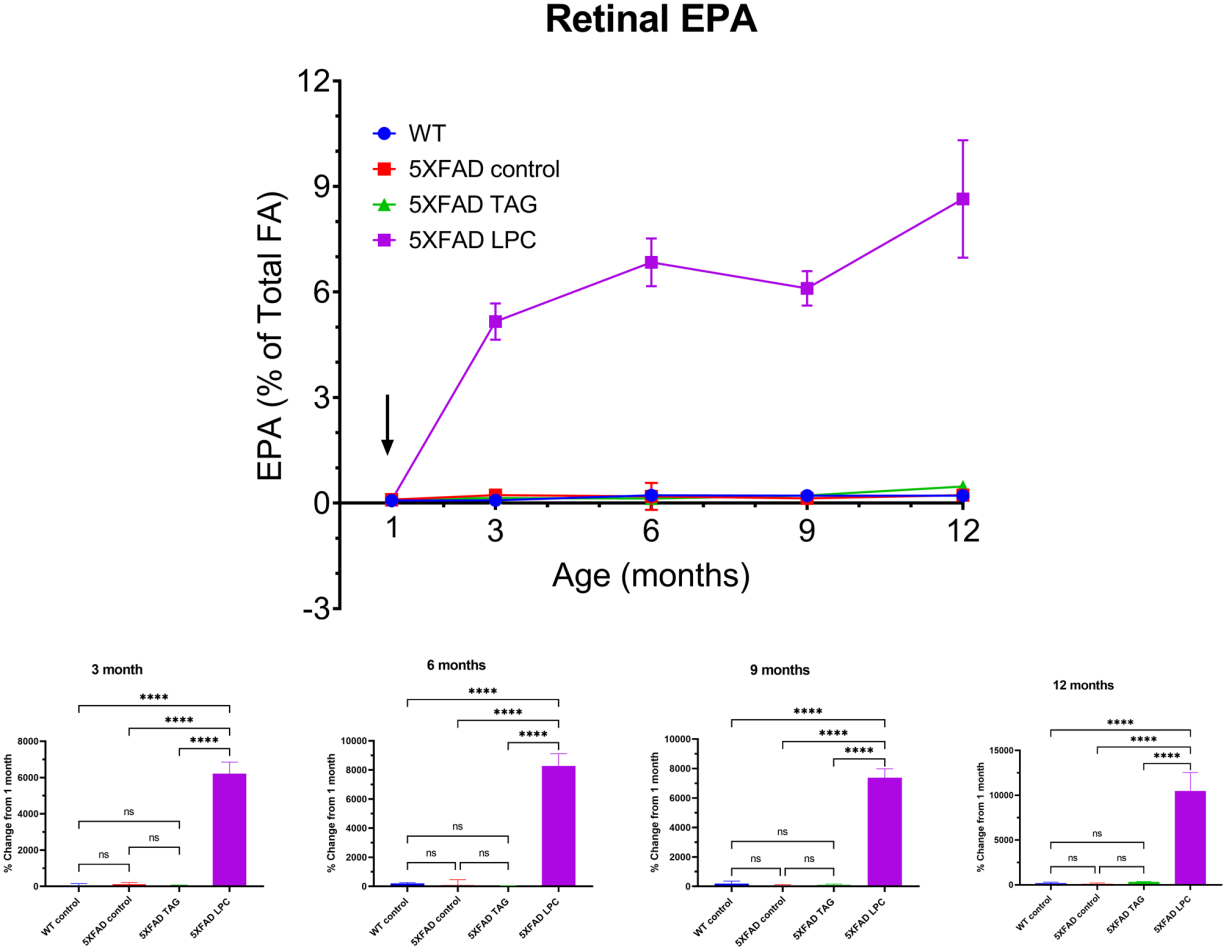
Time course of EPA incorporation into retinal lipids. The experimental conditions and the statistical analyses were the same as described under Fig. 1.

The time course of changes in retinal EPA levels are shown in Fig. 2. There is very little EPA in the retina of either WT or 5XFAD mice at 1 month, as has been reported for the adult mice^34–36^. Treatment with TAG-omega 3 diet did not increase the retinal EPA, whereas treatment with the LPC-omega 3 diet increased it by about 100-fold, as shown by us previously in the WT mice^35^. By 12 months of age, the EPA content of retina reached 8.6% of the total fatty acids in LPC-fed group, whereas it remained close to basal level (0.5%) in the TAG-fed group. This shows that only the LPC form of EPA can significantly increase retinal EPA, whereas the retinal DHA can be increased by both LPC and TAG molecular forms, although the latter is less efficient. At 12 months, the increase in DHA and EPA occurred mainly at the expense of 18:0, 18:1 (n-9), and 20:4 in the LPC-group, whereas the increase in the TAG group was mainly due to a decrease in 16:0 (Table 1, Supplemental data).

**Figure 3:**
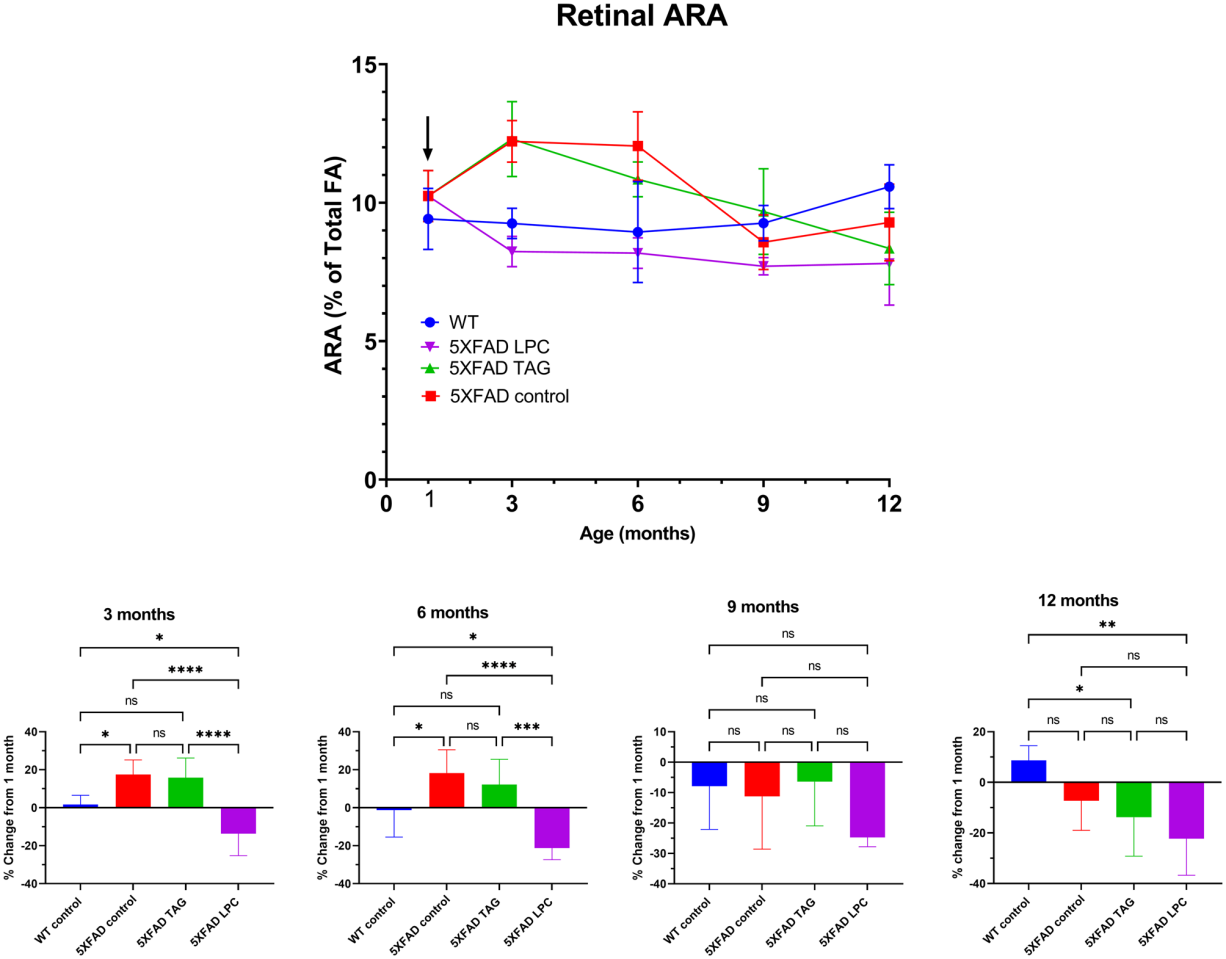
Time course of ARA incorporation into retinal lipids. The experimental conditions and the statistical analyses were the same as described under Fig. 1.

The time course of changes in ARA, the major omega 6 fatty acid in the retina are shown in Fig. 3. The retinal ARA was higher in the 5XFAD mice compared to the WT mice at 1 month, although the difference was not statistically significant. By 3 months, however, it increased further in the 5XFAD mice fed control diet, as well as the TAG diet, but was decreased in the LPC diet fed group. These results suggest that DHA and EPA from the LPC diet replaced ARA in retinal phospholipids, while the TAG diet had no effect, although both diets had similar amounts of EPA and DHA. After 6 months, however, the ARA level decreased in the control 5XFAD also, possibly because of increased oxidative stress. Consequently, by 12 months the ARA level was lower in all groups of 5XFAD mice, compared to the WT mice. At this time point neither diet decreased the ARA content significantly, although the decrease was greater with the LPC diet (Fig. 3, lower panel). It may be pointed out that a striking difference between the effects of LPC diet and TAG diet was the enrichment of retinal EPA by the LPC but not by TAG. Since the ARA levels decreased only with the LPC diet, these results suggest that EPA from dietary LPC specifically replaces ARA, whereas DHA replaces predominantly 18:0, 18:1, and 16:0, the major fatty acids in the retinal phospholipids.

### Retinal amyloid levels

**Figure 4:**
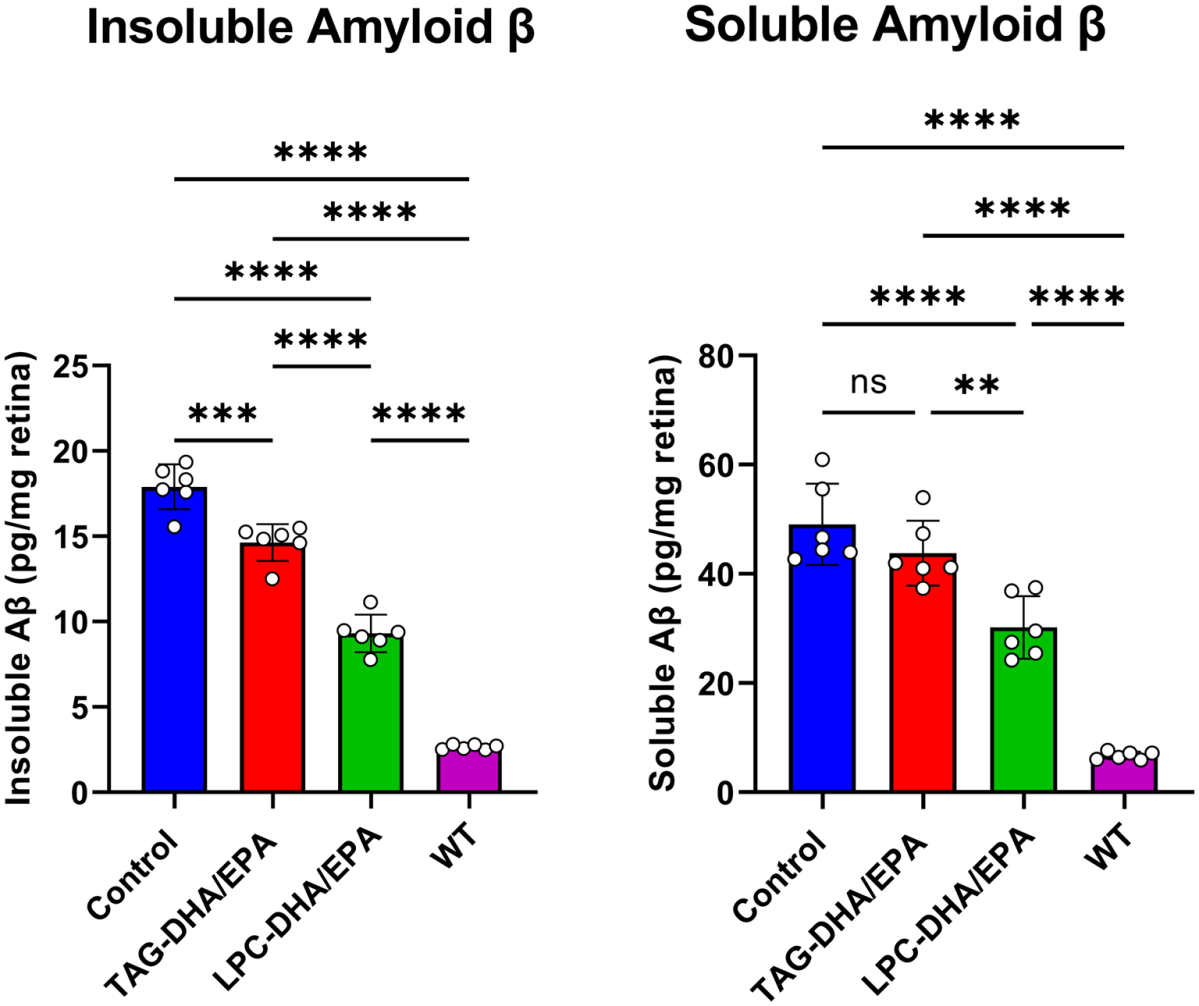
Analysis of amyloid β levels in retina. Soluble and insoluble amyloid β levels in the retina extracts were quantified using commercial ELISA kit (KMB3441; Invitrogen, Waltham, MA, USA) as described in the text. Values shown are mean ± SD of 6 samples for each group. Statistical significance was determined by ANOVA with Prism (Graphpad) software. The statistical symbols are the same as in Fig. 1.

Results of Fig. 4 show the effect of the diets on the soluble and insoluble amyloid β in retina at the age of 3 months. WT mice had very low levels of both soluble and insoluble amyloid β, whereas the 5XFAD mice showed a sevenfold increase in both forms of amyloid β. The results of Lim et al.^37^ also show a significant amyloid deposition in 5XFAD mice at the age of 6 months. This is in contrast to the APPswe/PS1Δ9 mice which did not show amyloid deposits in retina until the age of 12 months^38^. The TAG diet decreased the insoluble amyloid β levels by 17% and the soluble amyloid β by 11% in 5XFAD mice, whereas the LPC diet decreased the insoluble amyloid β by 49% and the soluble amyloid β by 39%. These results show that enriching retinal DHA and EPA by the LPC diets at early age significantly inhibits the deposition of amyloid β in retina. Similar results were obtained in the brains of the 5XFAD mice at this age (unpublished data).

### Effect of the TAG and LPC diets on retinal function

**Figure 5:**
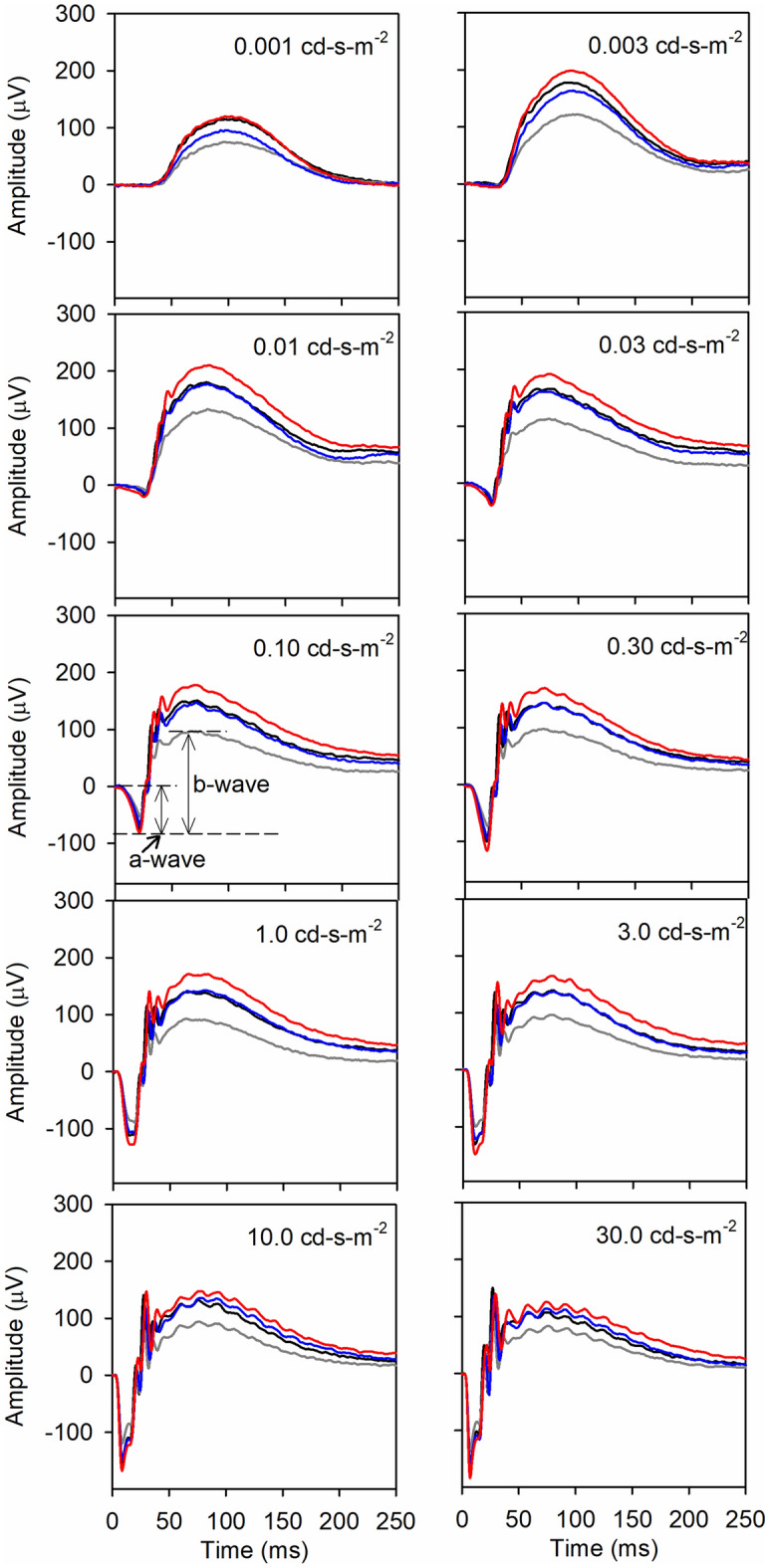
ERG waveforms measured at 3 months. Mice were dark-adapted for 2 h, and the ERG measurements were performed under dim red illumination as described in Methods. Each panel shows the response elicited at a different flash luminance level. The curves shown are averages of 15 mice in each group. The a-wave is measured from the baseline (0 µV) to the trough of the response (indicative of photoreceptor function), whereas the b-wave was measured from the trough of the a-wave to the peak of b-wave (indicative of bipolar activity). Black line: WT control; Gray line: 5XFAD control; Blue line: 5XFAD TAG diet; Red line: 5XFAD LPC-diet.

In order to determine the effect of DHA enrichment on retinal function, we measured ERG responses at 3 months of age (2 months of dietary treatment) since previous studies showed a significant defect in the ERG of 5XFAD mice at this age^39^. The mean ERG waveforms for the four groups of mice (WT control, 5XFAD control, 5XFAD TAG, and 5XFAD LPC groups, n = 15 per group) obtained under dark adapted conditions are shown in Fig. 5. Each panel shows the response of all the groups at increasing flash luminance (from 0.001 to 30 cd-s-m^−2^). The a-wave (the negative trough at the beginning of the graph) represents mainly the photoreceptor function, whereas the b-wave, which is measured from the a-wave trough to the b-wave peak is indicative of the response predominantly from the bipolar cells (illustrated in the 3rd panel on the left side). The overall pattern shows that the b-wave is clearly reduced in the 5XFAD mice on control diet (gray line), compared to their WT littermates (black line) on the same diet. Feeding the TAG diet to the 5XFAD mice for 2 months (blue line) improved the response substantially, approaching the level of WT mice. Feeding the LPC diet (red line), however, increased the response higher than even the WT group.

**Figure 6:**
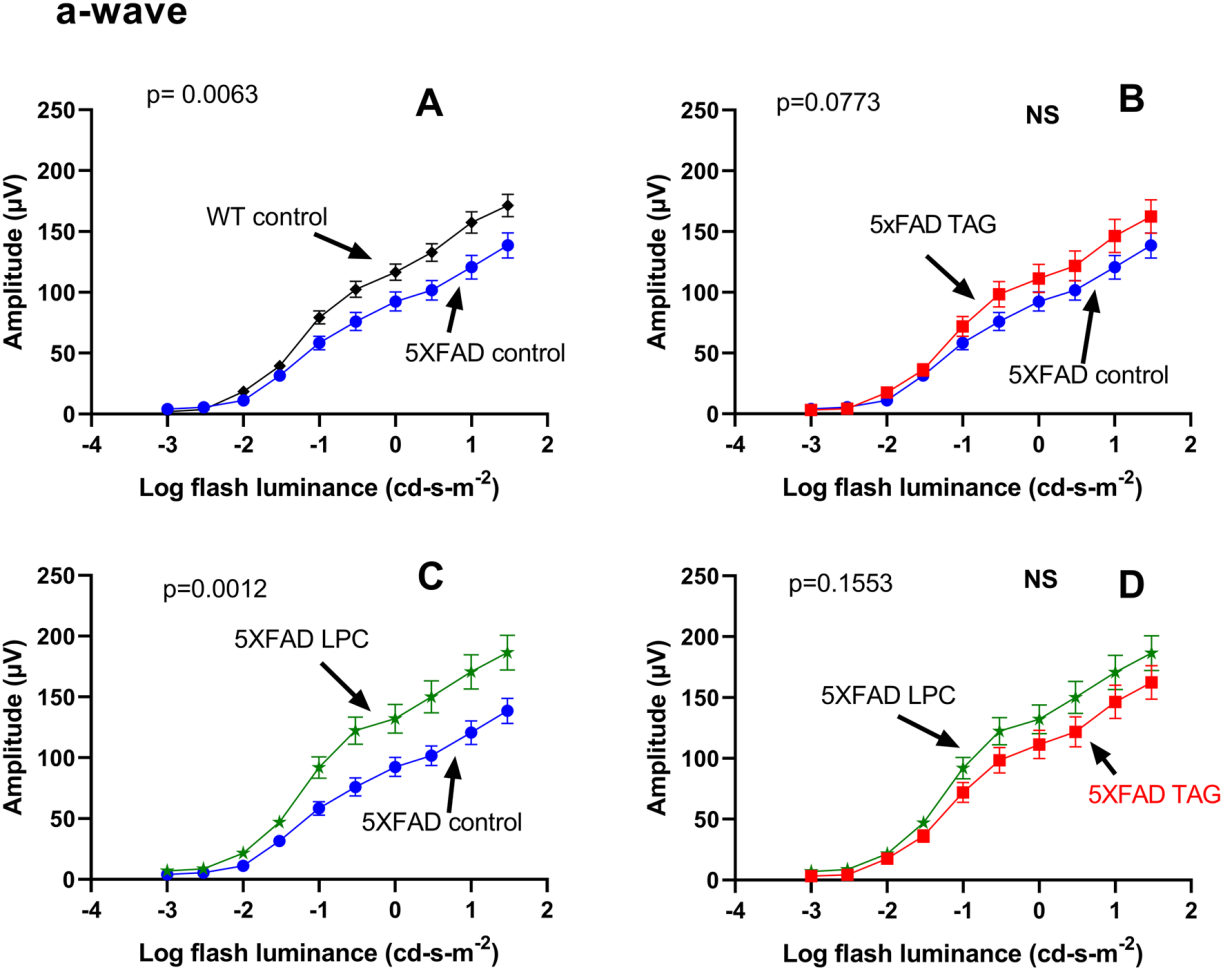
Mean a-wave amplitudes at increasing flash luminance levels. The a-wave amplitudes were extracted and the diet groups compared using the GEE model (n = 15 for each group). Pairwise comparisons are shown for clarity. Mean values ± SEM are plotted for various luminance levels, and the *p* values were corrected for multiple comparisons by the Bonferroni method, with the *p* values < 0.0083 (0.05/6) being considered as significant.

To further examine the differences between the groups, individual waveforms were extracted and compared pairwise, as shown for the a-wave in Fig. 6. Compared to their WT littermates, the 5XFAD mice exhibited significantly lower a-wave amplitude (A) (β = 7.45, p = 0.0063). Treatment with the TAG diet improved the response at all flash levels (B), but was not statistically significant (β = 3.12, *p* = 0.0773), whereas treatment with the LPC diet (C) significantly improved the response at all flash points (β = 10.53, *p* = 0.0012). Although the LPC treatment showed larger a-wave response than TAG treatment at all flash levels (D), the differences between the two diets was not statistically significant (β = 2.02, *p* = 0.1553).

**Figure 7:**
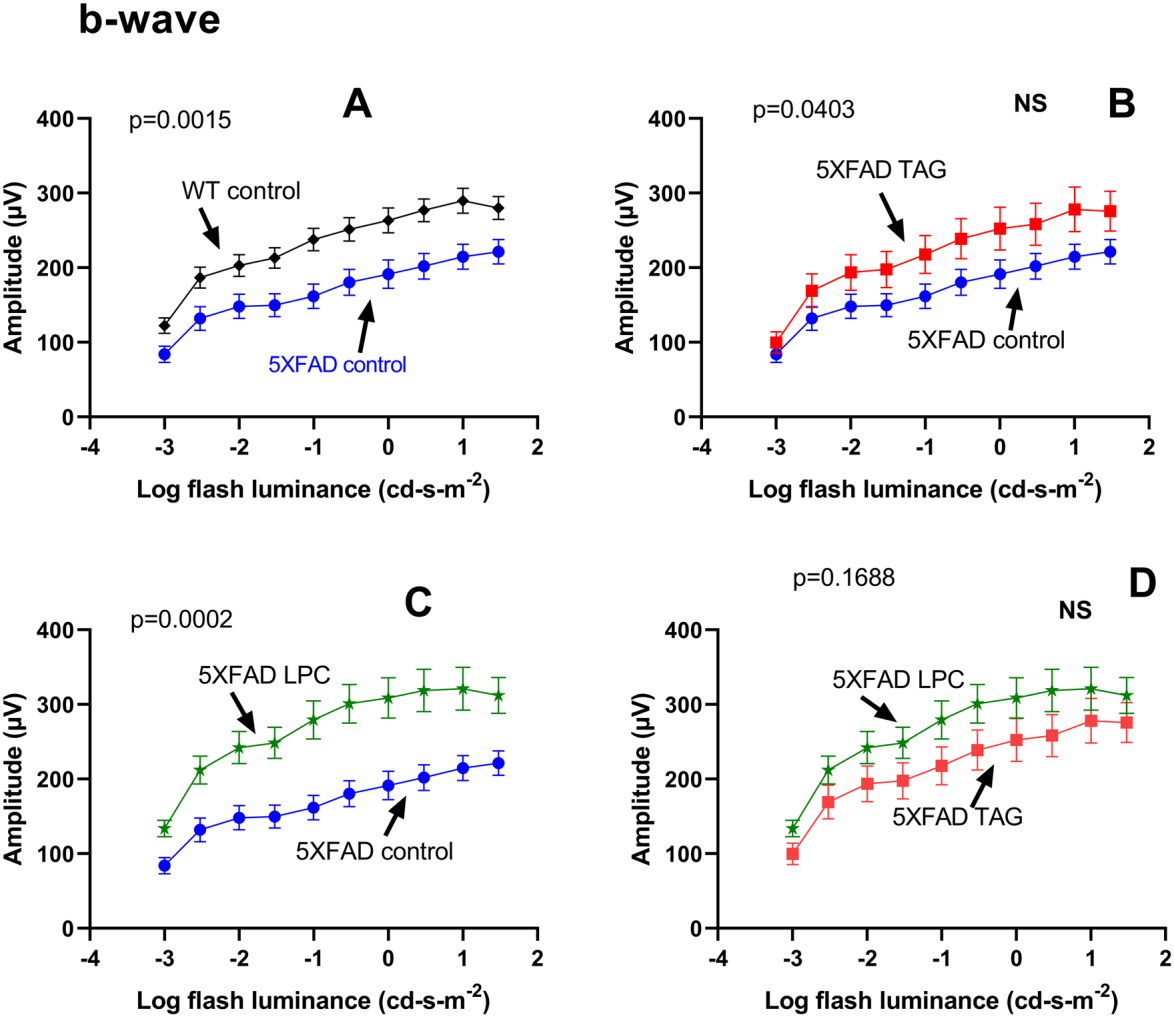
Mean b-wave amplitudes at increasing flash luminance levels. The extracted b-wave amplitudes were calculated and compared employing the GEE model. Values shown are mean values ± SEM, n = 15 in each group. The p values were corrected for multiple comparisons by the Bonferroni method, and the *p* values < 0.0083 were considered significant.

Results of Fig. 7 show the pairwise comparison of the extracted data for the b-wave. The results show a significantly diminished b-wave response in 5XFAD mice on control diet, compared to their WT littermates (A) (β = 10.09, *p* = 0.0015). Treatment of the 5XFAD mice with TAG diet (B) increased the b-wave amplitude, but this improvement was not statistically significant after the Bonferroni correction for multiple comparisons (β = 4.21, *p* = 0.0403). On the other hand, treatment with the LPC diet (C) significantly increased the b-wave amplitude (β = 13.87, *p* = 0.0002). The differences between the TAG group and the LPC group, however, were not statistically significant (β = 1.89, *p* = 0.1688) (D). These results show that, similar to the a-wave response, both treatments improve the b-wave response, but only the LPC treatment showed a statistically significant effect.

**Figure 8:**
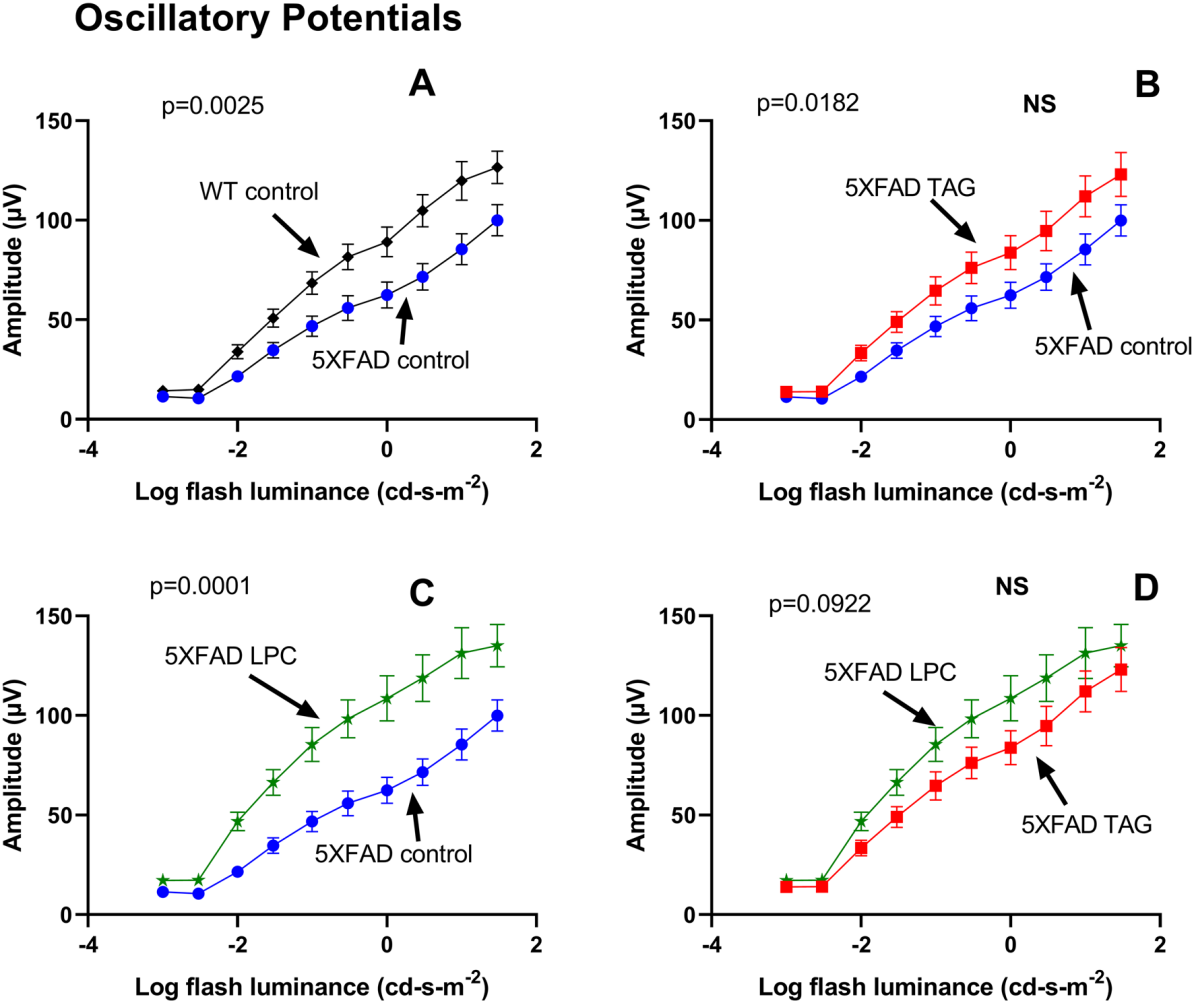
Oscillatory potentials (OP) at increasing flash luminance levels. The OP were extracted for each luminance level using the band pass filter (70–300 Hz), and the pairwise comparisons are shown. Values shown are means ± SEM, n = 15 in each group. The *p* values were corrected for multiple comparisons with the Bonferroni method, and the *p* values < 0.0083 were considered significant.

The oscillatory potentials (OP), primarily generated by amacrine cells, were extracted for each flash level using a band pass filter (70–300 Hz). The pairwise comparisons of the OP amplitude between the four groups are shown in Fig. 8. The GEE model showed a significant reduction in the OP amplitude in the 5XFAD mice, compared to the WT mice (β = 9.15. *p* = 0.0025) (A). Treatment of 5XFAD mice with TAG diet (B) showed some, but not statistically significant, improvement (β = 5.57, *p* = 0.0182). However, treatment with LPC diet (C) resulted in a more robust improvement (β = 14.39, *p* = 0.0001). The differences between the TAG treatment and LPC treatment, however, did not reach significance (β = 9.25, *p* = 0.0922) (D).

The implicit times for a-wave, b-wave, and OPs were, in general, similar for all groups of mice with only a few significant differences at isolated flash levels (results not shown). In summary, the results showed a consistent decline in the amplitudes of a-wave, b-wave, and OPs in the 5XFAD mice, compared to their WT littermates, a partial but insignificant reversal by the TAG diet, and a more robust and significant improvement with the LPC diet.

### OCT measurements

In addition to the ERG measurements, we measured the retinal thickness in the four groups of mice at the age of 3 months by optical coherence tomography (OCT), as described in Methods. There was a tendency for the 5XFAD mice to show decreased retinal thickness compared to the WT mice, but the difference was not statistically significant (Supplemental Fig. 2). This result suggests that functional impairments may occur before morphological changes such as cell loss or atrophy. The LPC diet showed a greater increase in thickness compared to the TAG diet, although the differences were not significant. A previous study showed a significant reduction in retinal thickness and alteration in neurovasculature in 6-month-old 5XFAD mice^40^. Therefore, additional studies with larger sample sizes and longer treatment periods is necessary to further validate these findings.

## Discussion

The results presented here show for the first time that the retinopathy associated with AD can be ameliorated significantly by increasing the retinal DHA through dietary LPC-EPA/DHA. There was also a significant reduction in both soluble and insoluble amyloid β in the retina by LPC-EPA/DHA. Although DHA has been shown to decrease the amyloid levels in the brain^41^, the effect of DHA on retinal amyloid levels has not been reported. Accumulation of amyloid β in retina induces several pathological changes, including neuronal and synaptic toxicity, mitochondrial dysfunction, and apoptosis of ganglion cells^42^. In addition to AD, retinal deposition of amyloid β is known to occur in other retinal diseases such as glaucoma and age related macular degeneration^42^. It would be interest to investigate if dietary LPC-EPA/DHA can also decrease the amyloid levels and improve the pathology in these diseases.

There are several common features between retina and the brain, including their common embryonic origin, shared neurochemistry including high DHA levels, similar properties of the blood–brain and blood-retina barriers, and common pathophysiology such as amyloid β accumulation in AD. Although the decline in memory and cognitive functions are the most prominent features of AD, abnormalities of vision may actually appear earlier than the cognitive impairments^3,5^. Because of this, and its non-invasive accessibility, retina is being increasingly used as a surrogate for the brain for the early detection of AD, and for evaluating preventive or therapeutic interventions. The beneficial effects of LPC-EPA/DHA on retinal composition and function, as shown here, could therefore reflect similar benefits on the brain function.

A novel finding of the study is that the retinal DHA levels were significantly decreased in 5XFAD mice even at the early age of 1 month, suggesting either a defect in the acquisition of DHA during gestation and lactation periods, or an increased loss of DHA because of oxidative stress in AD. Although one study reported decreased levels of Mfsd2a in the blood of AD patients, the expression of Mfsd2a in the (post-mortem) brain was found to be unaffected^43^. Interestingly, another study reported that in Mfsd2a knockout mice which showed a 50% reduction in retinal DHA, there was no impairment of visual signal transduction, although there was a reduction in the number of photoreceptor cells^44^. This is in contrast to several other studies which showed that retinal DHA plays an important role in maintaining proper visual function^45–47^. It is likely that the depletion DHA by congenital deficiency of Mfsd2a is not comparable to DHA deficiency achieved by dietary manipulation or due to oxidative stress. While the expression of Mfsd2a, as well as its protein levels have been reported to be normal in 5XFAD mice^48^, there is strong evidence for increased oxidative stress in 5XFAD mice^49,50^, which could lead to loss of retinal DHA.

A major difference between the effects of TAG diet and the LPC-diet is the incorporation of dietary EPA into the retinal lipids. Dietary LPC-EPA, but not the TAG-EPA increases the EPA level in the retina. The steady state level of EPA in brain and retina is very low, and previous studies showed that dietary EPA does not increase this level significantly^51–53^, although one study reported an increase in retinal EPA in aged rats after feeding a very high dose omega 3 FA as fish oil^34^. While it was earlier suggested that this low level of EPA in the brain is due to its rapid metabolism and oxidation^54^, our previous^35^ and current studies show that the molecular carrier of dietary EPA is the determining factor in its accretion and retention by brain and retina. Our results also suggest that EPA specifically replaces ARA from the retinal lipids. Thus, whereas the retinal EPA content increased by 100-fold from the baseline at 12 months (Fig. 2), the EPA/ARA ratio increased by 150-fold (Supplemental Fig. 3) indicating reciprocal changes between these two fatty acids. In contrast to this, the DHA content of retina increased by about twofold (Fig. 1), and the DHA/ARA ratio also increased by the same magnitude (Supplemental Fig. 3) indicating no effect on ARA content by DHA. Despite its low steady state concentration in retina, EPA may actually play significant role in retinal health and function. For example, EPA is more anti-inflammatory compared to DHA because it competes more effectively with the pro-inflammatory ARA for incorporation into membrane lipids and as a substrate for cyclooxygenases and lipoxygenases that produce pro-inflammatory eicosanoids from ARA. It is also known that EPA is a preferred substrate for the synthesis of very long chain-PUFA^55^ which are uniquely concentrated in retina, and are vital to its function^56^. EPA is incorporated predominantly into the less abundant phosphatidylinositol (PI), whereas DHA is incorporated into PE and PC, the major phospholipids in retina^57^. Although they are minor components of total retinal phospholipids, PI and its phosphorylated forms play very important roles in photoreceptor function and neuroprotection^58^. Therefore, the enrichment of retinal EPA, in addition to DHA may be more beneficial for the retinal function than increasing DHA alone.

A limitation of our study is that we did not perform ERG studies, amyloid measurements and retinal thickness studies (OCT) at time points beyond 3 months. Therefore, the progression of the disease and its potential prevention by LPC-DHA/EPA could not be determined. Furthermore, the correlations between the DHA enrichment and the amyloid levels, as well as the mechanisms by which DHA/EPA enrichment of retina leads to protection against retinopathy need to be investigated in future studies.

Previous pre-clinical studies also reported beneficial effects of dietary DHA in other retinal diseases such as retinopathy of prematurity^59^, Stargardt disease^60^, age-associated degeneration^34^ and diabetic retinopathy^14^ with the TAG form of omega 3 FA, but the dosage used in those studies was too high to be of practical use in long term clinical setting. For example, the doses used in the studies with lactating mice^59^ and type 2 diabetic mice^14^ translate to about 14 g of omega 3 FA per day for a 70 kg human subject, based on the allometric calculations^61^, assuming an average weight of 25 g for mice, whereas the dose used in the Stargardt study^60^ is equivalent to 48 g omega 3 FA/day in humans. Even with these high doses, the enrichment of retinal DHA was minimal. This could account for the failure of the large clinical trials to detect meaningful benefits of the currently available DHA supplements at safe doses^17–19^. In comparison, the dosage of LPC-EPA/DHA used in this study is equivalent to only 280 mg of omega 3 fatty acids per day for a 70 kg human subject, but resulted in a 50% increase in retinal DHA and 100-fold increase in retinal EPA in 2 months. Therefore, dietary LPC-EPA/DHA is vastly superior to TAG-EPA/DHA in enriching retinal omega 3 FA, and could be potentially beneficial not only AD-related retinopathy but also other retinal diseases at clinically achievable doses.

## Supplementary Information

Supplementary Information.

## Data Availability

The data obtained and analyzed during this study are available from the corresponding authors on reasonable request.
