# Supplementary material for: Improvement of retinal function in Alzheimer disease-associated retinopathy by dietary lysophosphatidylcholine-EPA/DHA
Source: Sci Rep. 2023 Jun 6;13:9179. doi: 10.1038/s41598-023-36268-0 (PMC10244360; doi:10.1038/s41598-023-36268-0)

Supplemental Data

Dhavamani Sugasini, Jason C. Park, J. Jason McAnany, Tae-Hoon Kim, Guangying Ma, Xincheng Yao, Babu Antharavally, Anil Oroskar, Asha A. Oroskar, Brian T. Layden, and Papasani V. Subbaiah

Improvement of retinal function in Alzheimer disease-associated retinopathy by dietary lysophosphatidylcholine-EPA/DHA.

Table 1- Supplement

Retinal FA at 12 months of age

FA	WT control	5XFAD control	5XFAD TAG	5XFAD LPC
12:0	0.11 ± 0.07	0.10 ± 0.08	0.13 ± 0.12	0.05 ± 0.03
14:0	0.15 ± 0.10	0.15 ± 0.07	0.28 ± 0.48	0.55 ± 0.64
16:0	23.68 ± 1.95[a]	24.61 ± 2.20[a]	18.69 ± 8.54[b]	21.18 ± 4.17[a]
16:1 (n-7)	0.14 ± 0.13	0.15 ± 0.10	0.47 ± 0.20	0.36 ± 0.26
18:0	21.48 ± 0.53[a]	21.71 ± 0.50[a]	21.88 ± 3.05[a]	17.75 ± 2.20[b]
18:1(n-9)	19.58 ± 1.68[ab]	21.14 ± 3.53[ab]	23.98 ± 5.12[a]	17.58 ± 2.28[b]
18:1(n-7)	5.22 ± 0.84[ab]	5.58 ± 1.43[ab]	6.77 ± 2.34[a]	3.81 ± 0.38[b]
18:2 (n-6)	0.42 ± 0.66	0.71 ± 1.53	0.44 ± 0.74	0.11 ± 0.15
18:3 (n-6)	0.14 ± 0.16[a]	0.14 ± 0.09[a]	0.12 ± 0.13[a]	0.31 ± 0.26[b]
18:3 (n-3)	0.19 ± 0.09[a]	0.15 ± 0.08[a]	0.11 ± 0.05[a]	0.42 ± 0.24[b]
20:0	0.18 ± 0.08	0.18 ± 0.11	0.21 ± 0.08	0.16 ± 0.21
20:1 (n-9)	0.14 ± 0.09	0.13 ± 0.09	0.39 ± 0.79	0.13 ± 0.10
20:2 (n-6)	0.49 ± 0.39	0.29 ± 0.10	0.26 ± 0.22	0.21 ± 0.13
20:3 (n-6)	0.37 ± 0.27	0.47 ± 0.31	0.34 ± 0.32	0.18 ± 0.10
20:4 (n-6)	10.58 ± 0.79[a]	9.29 ± 1.38[a]	8.92 ± 0.94[a]	7.81 ± 1.50[b]
22:0	0.19 ± 0.14	0.16 ± 0.16	0.07 ± 0.02	0.08 ± 0.04
20:5 (n-3)	0.21 ± 0.08[a]	0.23 ± 0.09[a]	0.51 ± 0.05[a]	8.65 ± 1.67[b]
22:2(n-6)	0.37 ± 0.20	0.22 ± 0.19	0.45 ± 0.29	0.21 ± 0.16
22:4 (n-6)	0.13 ± 0.05	0.19 ± 0.08	1.47 ± 2.59	0.17 ± 0.24
22:5 (n-6)	0.22 ± 0.12	0.31 ± 0.28	0.06 ± 0.04	0.15 ± 0.11
22:5 (n-3)	0.23 ± 0.14	0.36 ± 0.24	0.12 ± 0.07	0.38 ± 0.20
22:6 (n-3)	13.69 ± 0.30[a]	11.64 ± 0.75[a]	16.64 ± 3.56[ab]	24.78 ± 0.99[c]

Values without common superscripts are significantly different from each other (ANOVA).

Suppl. Fig. 1. Sugasini et al

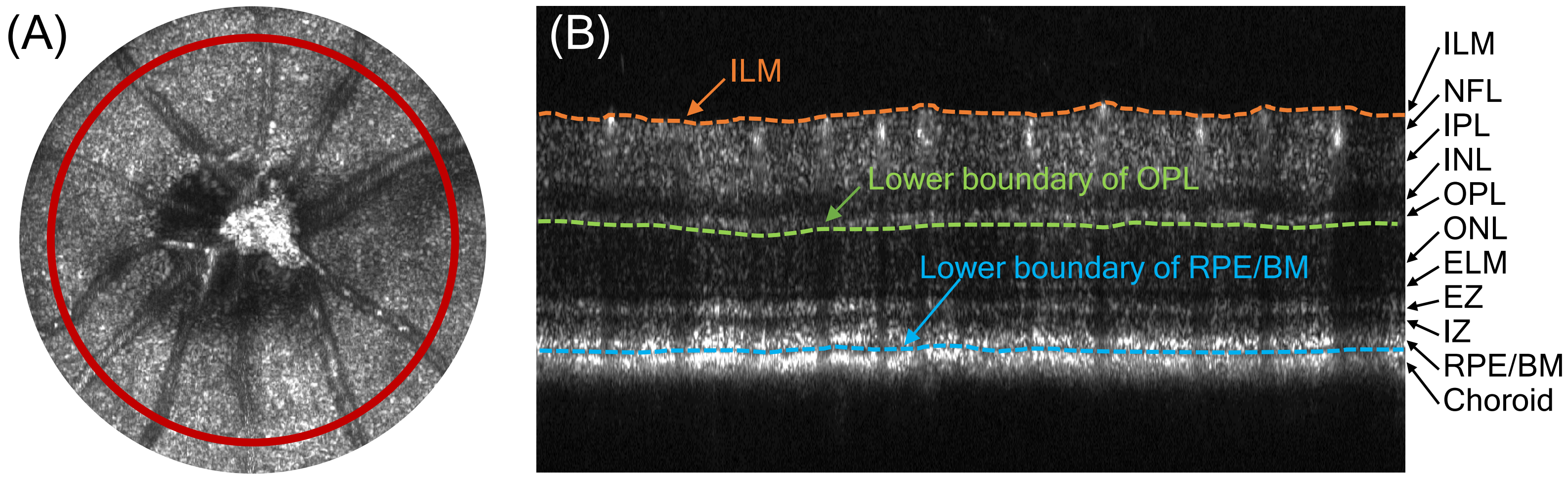

Supplemental Fig. 1. (A) Representative OCT enface image. (B) Representative circular B-scan, corresponding to the red circle in A. The inner and outer retina thicknesses were measured from ILM to the lower boundary of OPL (between the orange and green line), and from the lower boundary of OPL to the lower boundary of RPE/BM (between the green and blue line), respectively. ILM: inner limiting membrane; NFL: nerve fiber layer; IPL: inner plexiform layer; INL: inner nuclear layer; OPL: outer plexiform layer; ONL: outer nuclear layer; ELM: external limiting membrane; EZ: ellipsoid zone; IZ: interdigitation zone; RPE: retinal pigment epithelium; BM: Bruch membrane.

Suppl. Fig. 2. Sugasini et al

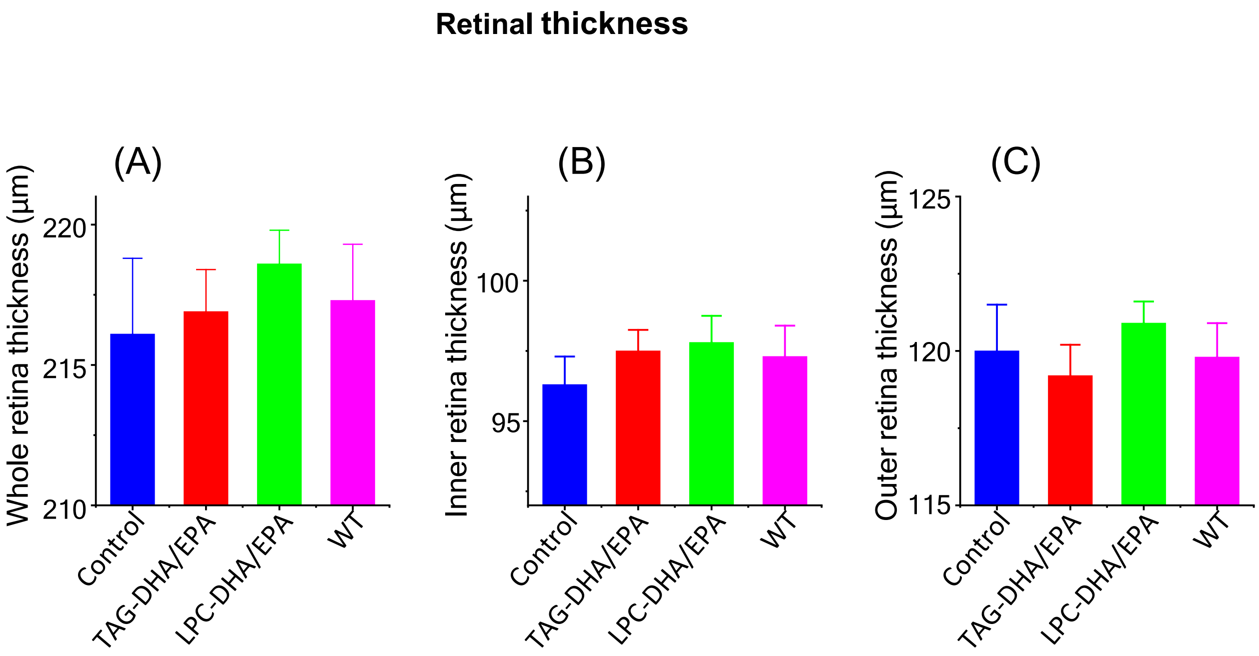

Supplemental Fig. 2. Retinal thickness was measured by OCT in 3 month old 5XFAD mice on the experimental diets and in 3 month old WT mice on control diet. A. Whole retina, B. Inner retina, C. Outer retina. (mean ± SEM, n=6 per group). The differences were not statistically significant.

Supplemental Fig. 3

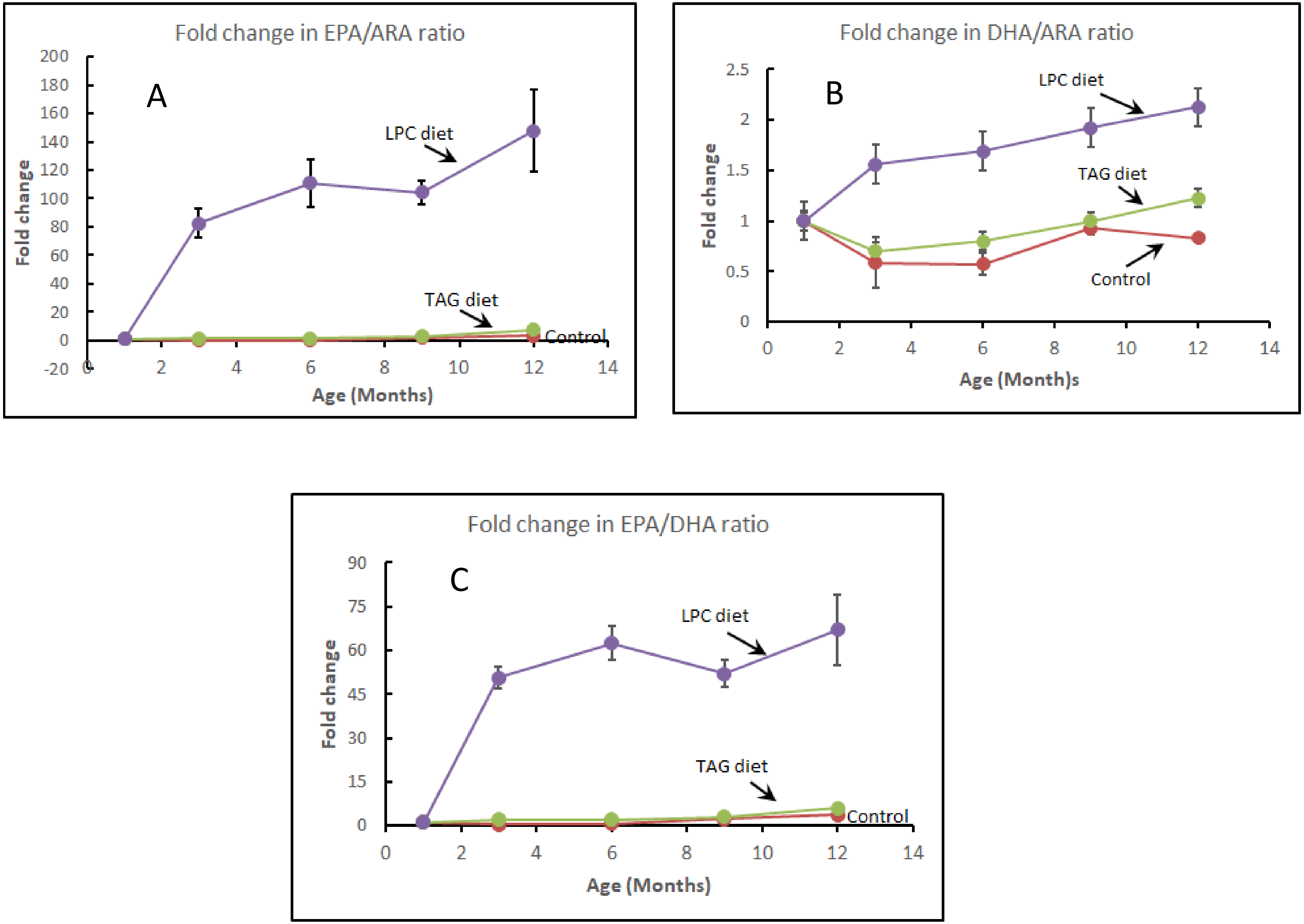

Supplement: Supplementary file 1 — Supplementary Information. [file 41598_2023_36268_MOESM1_ESM.pdf]
